# Correlation analysis between smoke exposure and serum neurofilament light chain in adults: a cross-sectional study

**DOI:** 10.1186/s12889-024-17811-8

**Published:** 2024-02-02

**Authors:** Ning Zhu, Jing Zhu, Shanhong Lin, Hang Yu, Chao Cao

**Affiliations:** 1 Department of Respiratory and Critical Care Medicine, Key Laboratory of Respiratory Disease of Ningbo, The First Affiliated Hospital of Ningbo University, 59 Liuting Road, 315010 Ningbo, Zhejiang China; 2 Department of Cardiology, The First Affiliated Hospital of University of Science and Technology of China (Anhui Provincial Hospital), Hefei, China; 3 Department of Ultrasound, The First Affiliated Hospital of Ningbo University, Ningbo, China

**Keywords:** Smoke exposure, Serum cotinine, Serum neurofilament light chain, Neurological function, NHANES

## Abstract

**Background:**

Smoke exposure is a prevalent and well-documented risk factor for various diseases across different organ systems. Serum neurofilament light chain (sNfL) has emerged as a promising biomarker for a multitude of nervous system disorders. However, there is a notable paucity of research exploring the associations between smoke exposure and sNfL levels.

**Methods:**

We conducted a comprehensive analysis of the National Health and Nutrition Examination Survey (NHANES) cross-sectional data spanning the years 2013 to 2014. Serum cotinine levels were classified into the following three groups: < 0.05, 0.05–2.99, and ≥ 3 ng/ml. Multiple linear regression models were employed to assess the relationships between serum cotinine levels and sNfL levels. Additionally, we utilized restricted cubic spline analyses to elucidate the potential nonlinear relationship between serum cotinine and sNfL levels.

**Results:**

A total of 2053 participants were included in our present research. Among these individuals, the mean age was 47.04 ± 15.32 years, and males accounted for 48.2% of the total study population. After adjusting the full model, serum cotinine was positively correlated with sNfl in the second group (β = 0.08, 95%CI 0.01–0.15) and in the highest concentration of serum cotinine (β = 0.10, 95%CI 0.01–0.19) compared to the group with the lowest serum cotinine concentrations. Current smokers, in comparison to non-smokers, exhibited a trend toward elevated sNfL levels (β = 0.07, 95%CI 0.01–0.13). Furthermore, subgroup analyses revealed interactions between serum cotinine levels and different age groups (*P* for interaction = 0.001) and gender stratification (P for interaction = 0.015) on sNfL levels.

**Conclusion:**

The study suggested that serum cotinine was significantly and positively associated with sNfl levels in adult participants. Furthermore, current smokers tend to exhibit elevated sNfL levels. This research sheds light on the potential implications of smoke exposure on neurological function impairment and underscores the importance of further exploration in this area.

## Introduction

Tobacco smoke exposure plays a pivotal role in the pathogenesis of a diverse spectrum of diseases, exerting endocrine-disrupting and neurotoxic effects [1]. Within tobacco smoke, numerous toxic and hazardous constituents, including cotinine, tar, polycyclic aromatic hydrocarbons (PAHs), formaldehyde, and other heavy metals, contribute to its detrimental impact [2]. Smoking stands as a predominant etiological factor in the development of various disorders, encompassing cardiovascular diseases (CVD), malignancies, chronic obstructive pulmonary disease, as well as neurobiological and neurocognitive abnormalities [3]. Cotinine, a major proximate metabolite of nicotine, serves as a reliable and sensitive indicator of cigarette smoke exposure [4]. Due to its longer half-life compared to its precursor, nicotine, cotinine is recognized as a distinct marker reflecting individuals’ levels of smoke exposure [5]. Smoking is recognized as a complex behavior associated with a spectrum of adverse health outcomes, including neurotoxicities. While nicotine has been a primary focus in understanding the neurobiological effects of smoking, it is essential to acknowledge that nicotine alone does not account for the entirety of smoking-related neurotoxicities. Tobacco smoke is a complex mixture containing various chemicals, each contributing to the overall neurotoxic profile [6]. Studies have suggested that PAHs [7], heavy metals [8], and formaldehyde [9] found in tobacco smoke have been implicated in diverse neurotoxic effects.

Toxicological responses to cigarette smoking are complex, influenced by a multitude of factors [10]. Genetic polymorphisms linked to biotransformation pathways shape individual responses, while various toxicants in cigarette smoke, such as cadmium and benzene, contribute to this intricate landscape [10]. Additionally, considering both occupational and non-occupational exposure scenarios broaden our understanding of the diverse contexts in which individuals encounter potential toxicants. Modifiable factors, including underlying metabolic diseases, alcohol intake, cigarette quantity and frequency, and duration of smoking, significantly contribute to variability in responses [10, 11]. Recognizing this interplay is crucial for a comprehensive appraisal of smoking’s health impact. Nutritional status is a pivotal determinant, influencing how the body processes cigarette smoke toxins [12]. This holistic perspective emphasizes the need to consider not only direct smoking effects but also the modulating influence of individual characteristics and environmental exposures.

Smoking exposure has been linked to neurotoxicity and its association with conditions such as traumatic brain injury, ischemic stroke, Alzheimer’s disease, and other neurological disorders is well-documented [6]. The neurotoxic effects of smoke exposure can be multifaceted and may involve multiple interrelated pathways. Cigarette smoke exposure induces oxidative stress in the body, leading to the production of reactive oxygen species (ROS), which in turn damages nerve cells [13]. Chronic inflammation is a hallmark of smoking-related health effects, and it may play a role in neurotoxicity by affecting the central nervous system [14]. Nicotine, a major component of tobacco, interacts with the neurotransmitter system and affects the release of dopamine and other neurotransmitters, which may have an effect on neurotoxicity [6, 15]. In addition, smoking induces DNA damage, and impairment of DNA repair mechanisms may lead to neurotoxic effects in the long term [16].

In routine clinical practice, serum neurofilament light chain (sNfL) has emerged as a promising biomarker for a multitude of nervous system diseases [17]. Neurofilaments, comprising light, medium, and heavy chains, represent key cytoskeletal proteins expressed in neurons and released into cerebrospinal fluid (CSF) [18]. Due to its high specificity for the nervous system, alterations in NfL concentration have been reported in conditions such as Alzheimer’s disease, amyotrophic lateral sclerosis, and other neuropathologies [19, 20]. Furthermore, levels of NfL have been associated with acute neuronal injury, including ischemic stroke and cardiac arrest [21].

While NfL has garnered recognition as a biomarker for nervous system diseases, questions have arisen regarding its associations with common risk factors, particularly in the adult population. Recent studies have already highlighted potential influences on NfL levels stemming from factors such as age, renal function, and body mass index (BMI) [22]. Tobacco smoke exposure ranks among the most prevalent risk factors for neurological diseases [23]. However, there is a notable paucity of research exploring the associations between smoke exposure and sNfL levels. Therefore, based on the NHANES cross-sectional data from 2013 to 2014, we aimed to elucidate the association between serum cotinine, cigarette smoking, and sNfL levels.

## Materials and methods

### Study design and population

The National Health and Nutrition Examination Survey (NHANES) is an ongoing cross-sectional survey conducted by the National Center for Health Statistics (NCHS) within the Centers for Disease Control and Prevention (CDC). Comprehensive information pertaining to NHANES operation manuals, estimation procedures, consent documents, and data analytic guidelines can be accessed through the NHANES website (https://wwwn.cdc.gov/nchs/nhanes/). NHANES has received ethical approval from the NCHS Ethics Review Board, and all study participants provided informed consent.

**Fig. 1:**
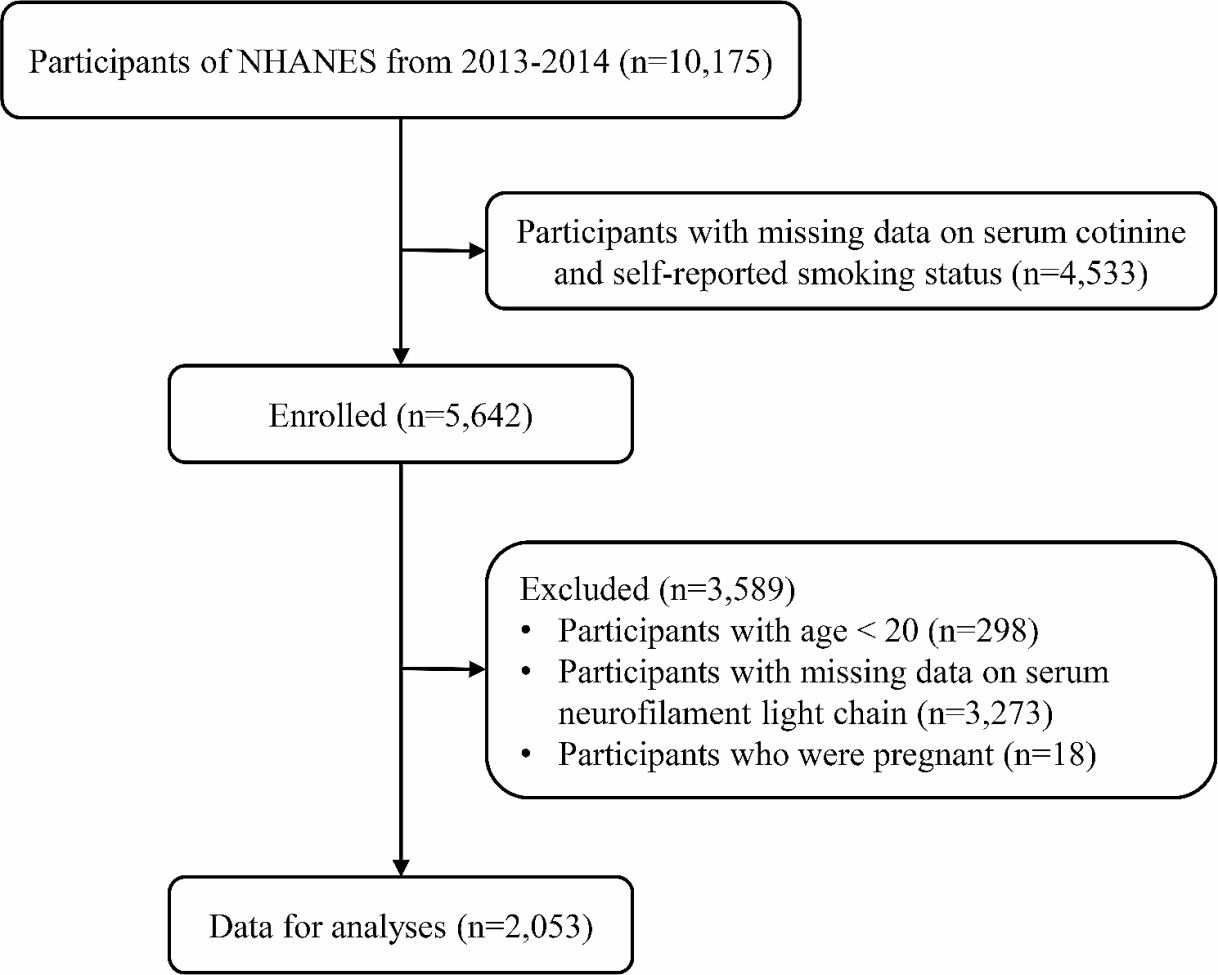
Flowchart of participants

Due to the limited availability of sNfL data, our analysis focused exclusively on the NHANES 2013–2014 cycle. Out of the original 10,175 participants involved in this survey, 4,533 were excluded due to missing data pertaining to serum cotinine levels and self-reported smoking status. Additionally, we excluded participants who were younger than 20 years of age (*n* = 298) and those who were pregnant (*n* = 18). Subsequently, we further excluded individuals for whom data on sNfL were not available (*n* = 3,273). This culminated in the inclusion of a total of 2,053 participants in our current research, as illustrated in Fig. 1.

### Measurements of smoke exposure

Serum cotinine specimens were obtained through venipuncture and subjected to analysis utilizing an isotope-dilution high-performance liquid chromatography/atmospheric pressure chemical ionization tandem mass spectrometry (ID HPLC-APCI MS/MS) method. Detailed information on specific laboratory procedures can be accessed via the following link: (https://wwwn.cdc.gov/Nchs/Nhanes/2013-2014/COT_H.htm). The lower limit of detection (LLOD) for serum cotinine was 0.015 ng/ml, with 2,597 participants having serum cotinine levels below this threshold. Consistent with previous studies [24], serum cotinine levels were categorised into heavy smoke exposure (≥ 3 ng/ml), light smoke exposure (0.05–2.99 ng/ml) and no smoke exposure (< 0.05 ng/ml) groups.

During the self-reported interviews, individuals who reported having smoked fewer than 100 cigarettes in their lifetime were categorized as nonsmokers. Participants who had smoked more than 100 cigarettes but had quit were designated as former smokers, while those who were currently smoking were classified as current smokers.

### Measurements of sNfL

Consistent with the methodology of previous studies [25], sNfL levels were quantified utilizing a highly sensitive immunoassay specifically designed for sNfL by Siemens Healthineers. This immunoassay employs acridinium ester (AE) chemiluminescence and paramagnetic particles, allowing it to be seamlessly integrated into the existing high-throughput automated platform, Attelica. In this method, serum samples were initially incubated with AE-labeled antibodies that selectively bind to the NfL antigen. Subsequently, paramagnetic particles (PMP) coated with a capture antibody were introduced, forming complexes of the NfL antigen bound to AE-labeled antibodies and PMP. Unbound AE-labeled antibodies were then meticulously separated and removed. Following this step, an acid and base were added to initiate chemiluminescence, and the resulting light emission was measured using the fully automated Attelica immunoassay system.

To ensure the reliability and accuracy of analytical measurements, strict quality control/quality assurance procedures were implemented. This included the analysis of low, medium, and high concentration quality control (QC) samples during each 8-hour shift, along with additional replicate samples. The coefficient of variation (CV) and other relevant statistics were calculated to describe the QC samples across the spectrum of sNfL measures. Moreover, in our assay, the lower limit of detection (LLOD) for sNfL was 3.9 pg/ml, and 36 participants had serum sNfL below the lower limit of detection (LLOD, 3.9 pg/ml), which was replaced by the LLOD divided by the square root of two. In contrast, none of the 2085 participants in the NHANES 2013–2014 survey exceeded the upper limit of detection (ULOD, 500 pg/ml) for serum neurofilament protein. Details of the study methodology can be found at: https://wwwn.cdc.gov/Nchs/Nhanes/2013-2014/SSSNFL_H.htm.

### Assessment of other covariates

Comprehensive data regarding baseline demographic characteristics, lifestyle factors, and medical status were obtained through baseline questionnaires. Demographic characteristics encompassed the following variables: sex, age, race (categorized as non-Hispanic white, non-Hispanic black, and other race), educational attainment (divided into categories of below high school, high school, and above high school), and income level. The family poverty-to-income ratio (PIR), reflecting the ratio of family income, was employed to assess the economic status.

Lifestyle factors included: physical activity and BMI. Physical activity data were obtained from the Physical Activity Questionnaire within NHANES. In accordance with the questionnaire, individuals were stratified into three groups based on their physical activity level, namely “inactive,” “insufficiently active,” and “active.” BMI was calculated as the ratio of weight (in kilograms) to the square of height (in meters) [26].

Medical status variables included: estimated glomerular filtration rate (eGFR) and comorbidity. The eGFR was computed using the Chronic Kidney Disease Epidemiology Collaboration (CKD-EPI) equation [27]. The comorbidity index was represented as a count variable, assigning an additional point for each reported diagnosis of the following conditions: hypertension, diabetes mellitus, bronchial asthma, high cholesterol, depression, coronary artery disease, and congestive heart failure. BMI, eGFR, and the comorbidity index were treated as continuous variables in our analysis. Hypertension status was ascertained through questionnaires, based on whether participants had received a diagnosis of hypertension from a healthcare professional.

### Statistical analyses

We conducted all analyses following the recommended NHANES analysis guidelines, incorporating sample weights (WTSSNH2Y) and accounting for clustering and stratification. For further details on the utilization of sample weights and other analytical considerations, please refer to the “NHANES Analytic Guidelines” and the online NHANES tutorials available at the following website:https://wwwn.cdc.gov/nchs/nhanes/analyticguidelines.aspx.

Continuous variables that exhibited a normal distribution are presented as means with accompanying standard deviations (SD). Categorical variables are reported as counts with corresponding percentages. In order to approximate a normal distribution, continuous serum cotinine and sNfL levels underwent natural logarithm (ln) transformation.

Multiple linear regression models were utilized to assess the associations between smoking status, serum cotinine levels, and ln-transformed sNfL levels among the American adult population. β coefficients, accompanied by their corresponding 95% confidence intervals (CIs), were employed to quantify the relationships between these variables. We performed restricted cubic spline (RCS) analyses to characterize the non-linear relationship between serum cotinine and ln-transformed sNfL levels. The selection of knots was guided by minimizing the Akaike information criterion (AIC), and the non-linearity was assessed using a likelihood test.

Three models were constructed for this study with the following adjusted covariates: age (continuous), sex (male or female), race (Non-Hispanic White, Non-Hispanic Black, or Other), education level (below high school, high school, or above high school), family PIR (continuous), physical activity (categorized as inactive, insufficiently active, or active), BMI (continuous), eGFR (continuous), and comorbidity index (continuous), self-reported smoking status (categorized as never smoker, former smoker, or current smoker). The covariates identified in this study should be biologically plausible, based on our clinical expertise and experience, considering that there is a potential biological relationship between the primary variable and the outcome. Such a selection will increase the explanatory power of the results of the study. Moreover, we carefully review the relevant literature to understand the correction variables used in past studies [25]. The experience of other studies can provide clues about variable selection and ensure comparability of results. Finally, by calculating variance inflation factors (VIF) [28], care is taken to avoid correcting for too many variables, as this may lead to over-adjustment, generate spurious associations or mask the true relationship.

Furthermore, we conducted stratified analyses to elucidate the association between serum cotinine and ln-transformed sNfL levels within distinct subgroups. These subgroups were defined by age (20–39, 40–59, ≥ 60 years), sex (male, female), race (Non-Hispanic White, Other), smoking status (nonsmokers, former or current smokers), BMI (< 30, ≥ 30 kg/m^2^), and physical activity (inactive, insufficiently active, active). All statistical analyses were performed using R studio (the R foundation; version 4.0.1). Significance was determined at a two-sided *p*-value threshold of < 0.05.

## Results

### Baseline characteristics of participants

**Fig. 2:**
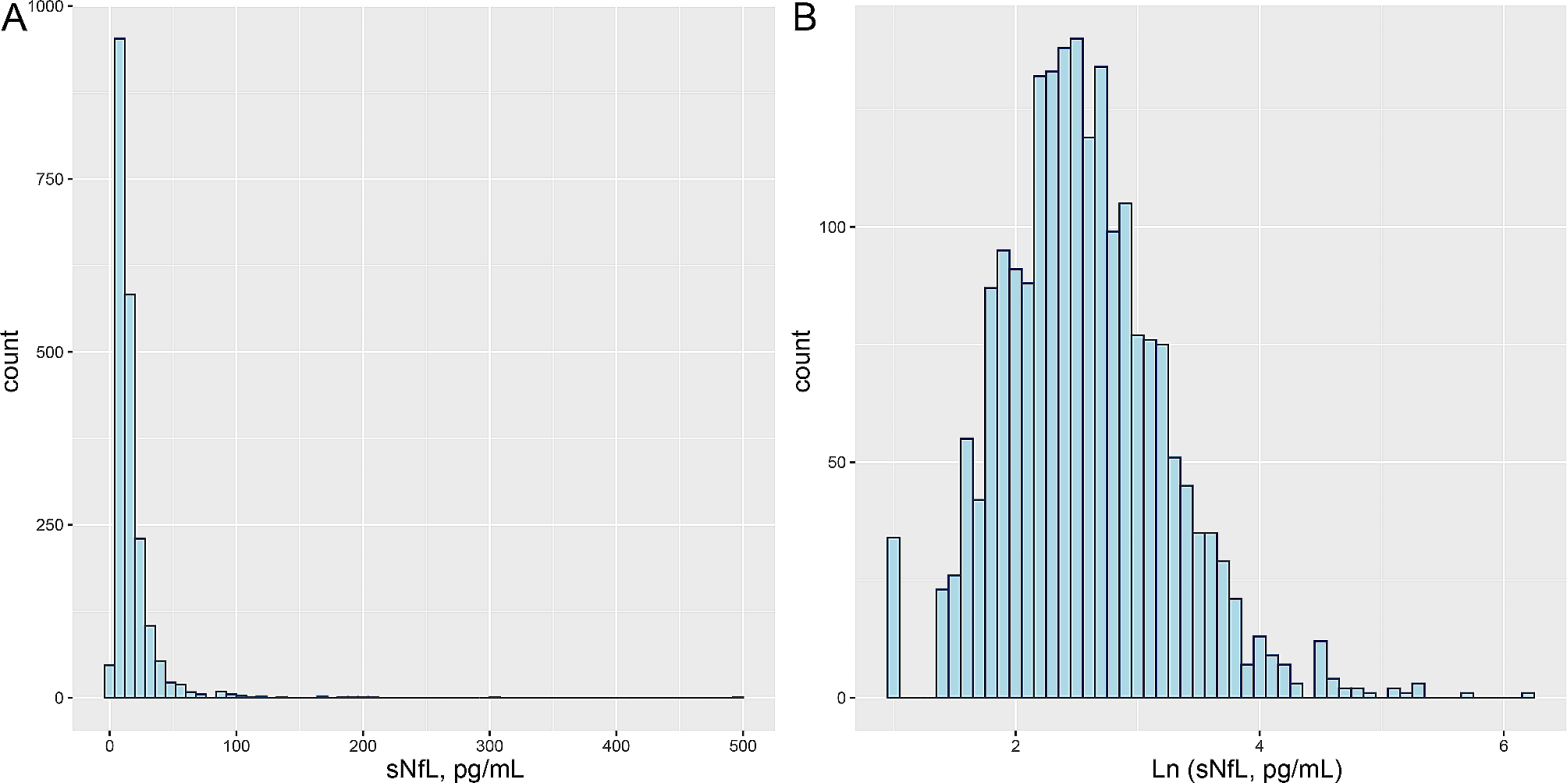
Distributions of serum neurofilament light chain (sNfL) concentrations (**A**); Ln-transformed distributions of sNfL levels (**B**)

Among the 2,053 individuals studied, there were 1,089 participants with serum cotinine levels below 0.05 ng/ml, 360 with serum cotinine levels ranging from 0.05 to 2.99 ng/ml, and 604 with serum cotinine levels equal to or greater than 3 ng/ml. Of these, 1,141 participants were nonsmokers, 458 were former smokers, and 452 were current smokers. The median serum cotinine level in the study population was 47.04 (15.32) ng/ml. Approximately 48.2% of the total study population were male. Ethnic distribution was as follows: 904 participants were Non-Hispanic White (44%), 367 were Non-Hispanic Black (17.9%), and 782 were of other racial backgrounds (38.1%). Furthermore, as illustrated in Fig. 2, the values of ln-transformed sNfL levels were found to deviate from a normal distribution, whereas the values of sNfL levels on the ln-transformed scale exhibited a log-normal distribution.

Table 1 provides a summary of the baseline characteristics of the study participants. In comparison to the 1,089 individuals in the lowest serum cotinine group, the 309 participants in the second group and the 604 participants in the third group were more likely to be younger (mean age 44.15 [16.17] years), have a higher family PIR (1.96 [0.76]), exhibit a higher BMI (30.71 [8.97] kg/m^2^), possess a higher eGFR (99.31 [21.89] ml/min/1.73 m^2^), and display a lower comorbidity index (0.86 [1.44]). In contrast, the 458 former smokers, compared to nonsmokers and current smokers, were more likely to be older (mean age 52.84 [14.18] years), male (61.4%), have the highest family PIR (2.22 [0.77]), highest BMI (29.96 [7.04] kg/m^2^), lowest eGFR (99.31 [21.89] ml/min/1.73 m2), and the highest comorbidity index (1.37 [1.83]).

**Table 1:** Baseline characteristics of participants in NHANES 2013–2014

Characteristics	Total	Cotinine category, %			P value	Self-reported smoking status, %			P value
		< 0.05 ng/mL	0.05–2.99 ng/mL	≥ 3 ng/mL		Nonsmoker	Former smoker	Current smoker	
Participants, n	2053	1089	360	604		1141	458	454	
Age, years	47.04 (15.32)	49.32 (14.86)	44.15 (16.17)	44.64 (14.97)	< 0.001	45.64 (15.50)	52.84 (14.18)	44.70 (14.52)	< 0.001
Male, %	990 (48.2)	464 (42.6)	172 (47.8)	354 (58.6)	< 0.001	468 (41.0)	281 (61.4)	241 (53.1)	< 0.001
Race/ethnicity, %					< 0.001				< 0.001
Non-Hispanic White	904 (44.0)	454 (41.7)	130 (36.1)	320 (53.0)		437 (38.3)	217 (47.4)	250 (55.1)	
Non-Hispanic Black	367 (17.9)	115 (10.6)	92 (25.6)	160 (26.5)		186 (16.3)	68 (14.8)	113 (24.9)	
Other race	782 (38.1)	520 (47.8)	138 (38.3)	124 (20.5)		518 (45.4)	173 (37.8)	91 (20.0)	
Education level, %					< 0.001				< 0.001
Below high school	451 (22.0)	187 (17.2)	93 (25.8)	171 (28.3)		210 (18.4)	105 (22.9)	136 (30.0)	
High school	429 (20.9)	165 (15.2)	88 (24.4)	176 (29.1)		206 (18.1)	91 (19.9)	132 (29.1)	
Above high school	1173 (57.1)	737 (67.7)	179 (49.7)	257 (42.5)		725 (63.5)	262 (57.2)	186 (41.0)	
Family PIR, %	2.14 (0.78)	2.34 (0.73)	1.96 (0.76)	1.87 (0.76)	< 0.001	2.22 (0.77)	2.23 (0.74)	1.83 (0.75)	< 0.001
Physical activity, %					0.129				0.108
Inactive	487 (23.7)	245 (22.5)	91 (25.3)	151 (25.0)		253 (22.2)	117 (25.5)	117 (25.8)	
Insufficiently active	673 (32.8)	385 (35.4)	108 (30.0)	180 (29.8)		385 (33.7)	158 (34.5)	130 (28.6)	
Active	893 (43.5)	459 (42.1)	161 (44.7)	273 (45.2)		503 (44.1)	183 (40.0)	207 (45.6)	
BMI, kg/m^2^	29.27 (7.39)	29.24 (6.90)	30.71 (8.97)	28.46 (7.06)	< 0.001	29.34 (7.55)	29.96 (7.04)	28.40 (7.23)	0.005
eGFR, ml/min/1.73 m^2^	96.10 (21.16)	93.61 (20.76)	99.31 (21.89)	98.67 (20.91)	< 0.001	97.26 (21.10)	90.86 (21.63)	98.45 (20.00)	< 0.001
Comorbidity index ^*^	0.99 (1.51)	1.02 (1.54)	0.86 (1.44)	1.01 (1.51)	0.195	0.81 (1.34)	1.37 (1.83)	1.05 (1.51)	< 0.001

Normally distributed continuous variables are described as means ± SDs. Categorical variables are presented as numbers (percentages). PIR, poverty income ratio; BMI, Body mass index; eGFR, estimated glomerular filtration rate. ^*^ A comorbidity index was coded as a count variable with an additional point for a reported diagnosis of each of the following: hypertension, diabetes mellitus, bronchial asthma, high cholesterol, depression, coronary artery disease, and congestive heart failure

### Association of smoking status, serum cotinine level and sNfL levels

To evaluate the association between smoking status, serum cotinine levels, and ln-transformed sNfL levels, we conducted multivariate linear regression analyses, utilizing either the participants with the lowest serum cotinine concentrations or nonsmokers as the reference category for these calculations (Table 2). In all three models, each adjusted for various covariates, a positive correlation was observed between serum cotinine and ln-transformed sNfL levels in the second group of serum cotinine (model 1: β = 0.08, 95%CI 0.01–0.15; model 2: β = 0.08, 95%CI 0.01–0.15; model 3: β = 0.08, 95%CI 0.01–0.15). This relationship between serum cotinine and ln-transformed sNfL levels remained robust in the group with the highest serum cotinine concentration (model 1: β = 0.11, 95%CI 0.05–0.17; model 2: β = 0.10, 95%CI 0.04–0.17; model 3: β = 0.10, 95%CI 0.01–0.19). When considering serum cotinine as a continuous variable, similar results were observed (model 1: β = 0.01, 95%CI 0.01–0.02; model 2: β = 0.01, 95%CI 0.01–0.02; model 3: β = 0.02, 95%CI 0.01–0.03).

**Table 2:** Association of smoking status, serum cotinine level and ln-transformed serum neurofilament light chain (sNfL) levels among adults in NHANES 2013–2014

	β (95% CI)		
	Model 1	Model 2	Model 3
Ln-transformed cotinine, ng/mL	0.01 (0.01, 0.02)	0.01 (0.01, 0.02)	0.02 (0.01, 0.03)
*P* value	< 0.001	< 0.001	0.001
Cotinine categories, %			
< 0.05 ng/mL	0.00 [Reference]	0.00 [Reference]	0.00 [Reference]
0.05–2.99 ng/mL	0.08 (0.01, 0.15)	0.08 (0.01, 0.15)	0.08 (0.01, 0.15)
≥ 3 ng/mL	0.11 (0.05, 0.17)	0.10 (0.04, 0.17)	0.10 (0.01, 0.19)
*P* for trend	< 0.001	< 0.001	0.008
Self-reported smoking status, %			
Nonsmokers	0.00 [Reference]	0.00 [Reference]	–
Former smokers	0.03(-0.04, 0.09)	0.03(-0.04, 0.09)	–
Current smokers	0.09(0.03, 0.16)	0.07(0.01, 0.13)	–
*P* for trend	0.006	0.034	–

Model 1 was adjusted as age (continuous), sex (male or female), and race (Non-Hispanic White, Non-Hispanic Black, or Other); Model 2 was adjusted as model 1 plus education level (below high school, high school, or above high school), family PIR (continuous), physical activity (inactive, insufficiently active, or active), BMI (continuous), eGFR (continuous) and comorbidity index (continuous); Model 3 was adjusted as model 2 plus self-reported smoking status (never smoker, former smoker, or current smoker)

Furthermore, in comparison to nonsmokers, current smokers exhibited a tendency towards higher sNfL levels (β = 0.09, 95%CI 0.03–0.16) in Model 1. After adjusting for education level, family PIR, physical activity, BMI, eGFR, and comorbidity index in Model 2, this relationship remained robust (β = 0.07, 95% CI 0.01–0.13).

### Non-linear analysis of the association of serum cotinine level and sNfL levels

**Fig. 3:**
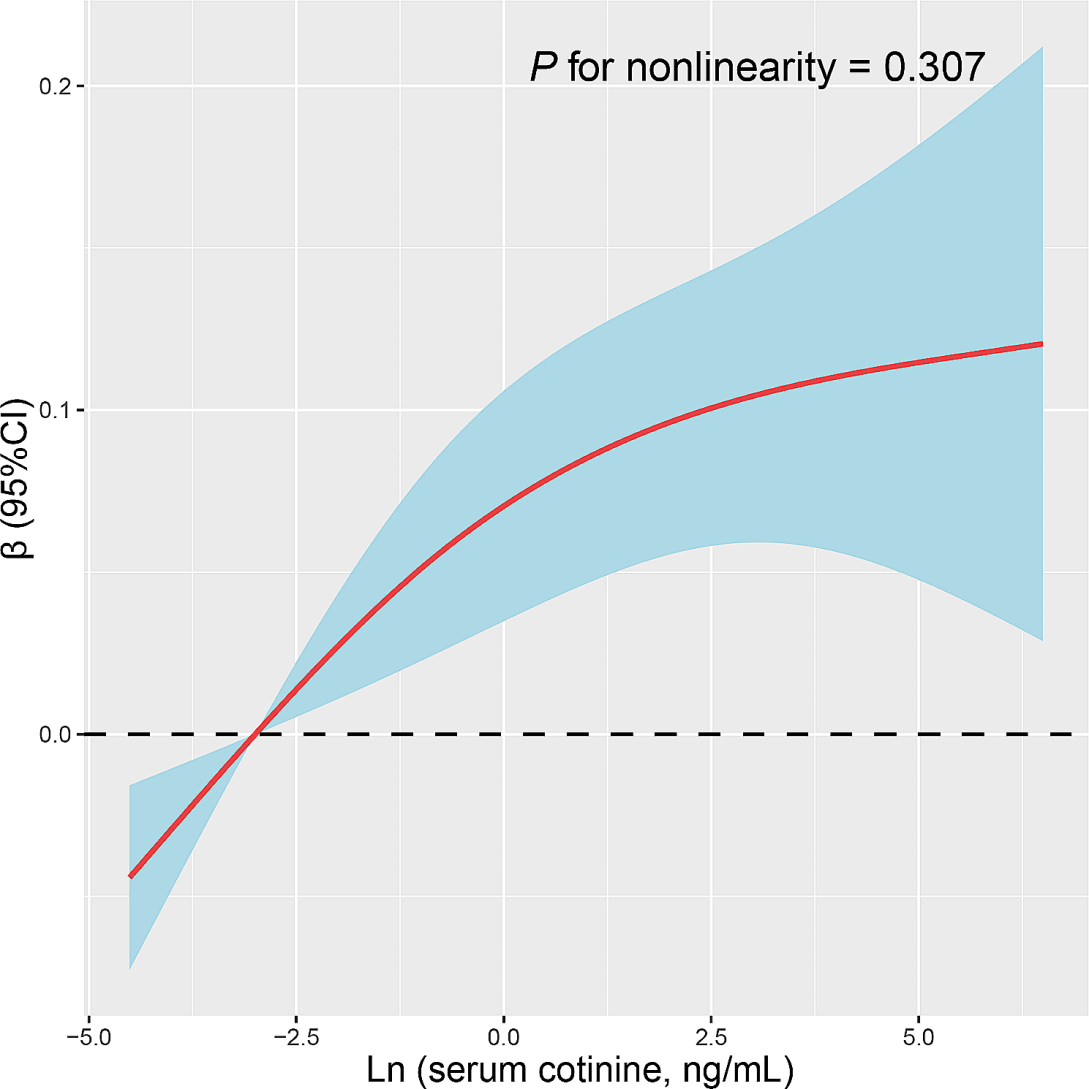
Restricted cubic spline (RCS) analysis with multivariate-adjusted associations between serum cotinine and serum neurofilament light chain (sNfL) levels in adults. Models are adjusted for age (continuous), sex (male or female), and race (non-Hispanic White, non-Hispanic Black, or Other), education level (below high school, high school, or above high school), family PIR (continuous), physical activity (inactive, insufficiently active, or active), BMI (continuous), eGFR (continuous), comorbidity index (continuous), and smoking status (never smoker, former smoker, or current smoker)

To explore the nonlinear relationship between serum cotinine levels and sNfL, we analysed this relationship using RCS regression. The results of multivariate linear regression with RCS are presented in Fig. 3. The results showed that a linear and positive correlation was observed between serum cotinine levels and sNfL levels (*P* for non-linearity = 0.307).

### Subgroup analysis and interaction analysis

Table 3 presents the associations between serum cotinine levels and ln-transformed sNfL levels in various subgroups stratified by different covariates. After adjusting for the comprehensive model, when compared to the group with the lowest serum cotinine concentrations, we observed that the serum cotinine in the group with the highest serum cotinine levels were positively correlated with ln-transformed sNfL levels in individuals older than 60 years (β = 0.31, 95%CI 0.12–0.50), males (β = 0.15, 95%CI 0.03–0.27), individuals of other racial backgrounds (β = 0.13, 95%CI 0.01–0.25), those with a BMI below 30 kg/m2 (β = 0.14, 95%CI 0.03–0.25), and those engaging in active physical activity (β = 0.18, 95%CI 0.06–0.31). Furthermore, differences in sNfL levels across various subgroups suggested that serum cotinine levels interacted with different age groups (*P* for interaction = 0.001) and gender stratification (*P* for interaction = 0.015), indicating additional effects of these specific subgroups on the associations between serum cotinine and sNfL levels.

**Table 3:** Stratified analyses of the associations between smoking status, serum cotinine level and ln-transformed serum neurofilament light chain (sNfL) levels according to smoking status among adults

Subgroups	N	Cotinine categories, ng/mL			P-t	P-int
		< 0.05	0.05–2.99	≥ 3		
Age, years						
20–39	700	0 [Reference]	0.14(0.03, 0.26)	0.09 (-0.06, 0.24)	0.076	0.001
40–59	811	0 [Reference]	0.01 (-0.12, 0.12)	-0.04 (-0.21, 0.12)	0.686	
≥ 60	542	0 [Reference]	0.02 (-0.11, 0.15)	0.31 (0.12, 0.50)	0.007	
Sex, %						
Male	542	0 [Reference]	0.11(0.00, 0.21)	0.15 (0.03, 0.27)	0.007	0.015
Female	1063	0 [Reference]	0.06(-0.03, 0.16)	0.01 (-0.14, 0.16)	0.379	
Race, %						
Non-Hispanic White	904	0 [Reference]	0.12(0.00, 0.23)	0.09 (-0.05, 0.23)	0.081	0.293
Other	1149	0 [Reference]	0.07(-0.01, 0.15)	0.13 (0.01, 0.25)	0.015	
Smoking status, %						
Nonsmokers	1141	0 [Reference]	0.10 (0.02, 0.19)	0.12 (-0.01, 0.25)	0.008	0.603
Former or current smokers	912	0 [Reference]	0.03 (-0.10, 0.16)	0.06 (-0.03, 0.16)	0.204	
BMI, kg/m^2^						
< 30	1275	0 [Reference]	0.09 (0.01, 0.18)	0.14 (0.03, 0.25)	0.004	0.061
≥ 30	778	0 [Reference]	0.06 (-0.05, 0.18)	0.03 (-0.13, 0.20)	0.432	
Physical activity, %						
Inactive	487	0 [Reference]	0.03 (-0.12, 0.18)	0.11 (-0.10, 0.32)	0.319	0.285
Insufficiently active	673	0 [Reference]	0.10 (-0.03, 0.23)	-0.06 (-0.23, 0.10)	0.939	
Active	893	0 [Reference]	0.08 (-0.02, 0.18)	0.18 (0.06, 0.31)	0.004	

Analyses were adjusted for covariates age (continuous), sex (male or female), and race (non-Hispanic White, non-Hispanic Black, or Other), education level (below high school, high school, or above high school), family PIR (continuous), physical activity (inactive, insufficiently active, or active), BMI (continuous), eGFR (continuous), comorbidity index (continuous), and smoking status (never smoker, former smoker, or current smoker) when they were not the strata variables. *p-t, p* for trend; *p-int*, p for interaction

## Discussion

In this cross-sectional study of US adults, linear regression models with comprehensive adjustments for confounding factors revealed a positive relationship between serum cotinine levels and ln-transformed sNfL levels. Cigarette smoke exposure was associated with elevated ln-transformed sNfL levels. Our results were substantiated through various stratified analyses, although we observed that different subgroups defined by age and sex exhibited varying associations between serum cotinine and sNfL. To our knowledge, our study is the first to investigate the relationship between serum cotinine and ln-transformed sNfL.

Previous studies have underscored the influence of various physiological and pathological factors on sNfL, complicating its interpretation as a specific biomarker for distinct disease factors [29]. Physiologically, sNfL levels are intricately linked to a range of factors that may influence its interpretation. For instance, pregnant participants were excluded from the study, recognizing the physiological increase in sNfL during pregnancy [30]. Furthermore, sNfL may be influenced by an increase in blood volume associated with BMI, suggesting a need for careful consideration of metabolic factors in the interpretation of results [31]. Thus, the impact of BMI was addressed through adjustment in the multivariate regression models, mitigating potential confounding effects related to changes in blood volume associated with BMI. From a pathological perspective, the lack of specificity of sNfL for particular disease factors complicates its utility as a specific biomarker. Neuronal damage, whether resulting from neurodegenerative diseases or head impacts during sports, can lead to an elevation in sNfL [32]. Moreover, the non-specificity of sNfL for the central nervous system extends to its occurrence with injury to the peripheral nervous system, raising questions about its specificity in neurologic disease contexts [33]. The presence of cardiovascular risk factors and the aging process introduces additional challenges, potentially causing subclinical damage due to silent ischemic events [34]. The multivariate regression model was employed to correct for the presence of CVD and related risk factors, including hypertension, diabetes, and hypercholesterolemia, to mitigate potential biases associated with neurologic and cardiovascular conditions. Overall, the intricate interplay of physiological and pathological factors underscores the complexity of interpreting sNfL levels. While it serves as a valuable biomarker for neuronal damage, its lack of specificity necessitates a cautious approach in associating changes in sNfL with specific disease outcomes [29, 34].

The relationship between serum cotinine and ln-transformed sNfL remains a subject of uncertainty. Some previous studies have indicated that smokers are more likely to have higher sNfL levels than nonsmokers [35]. Cigarette smoking is known to have detrimental effects on carcinogenic properties and brain neurobiology [3]. An array of cytotoxic compounds, such as nicotine, carbon monoxide, and nitrosamines, may directly impact the function of neuronal cells and tissues by promoting oxidative damage [3, 36]. Importantly, cigarette smoking does not occur in isolation, and it is often accompanied by other contributing factors in the context of cognitive function impairment. Previous research has highlighted that potential links between tobacco smoking and these contributors remain relatively unexplored. Our findings contribute to the growing body of evidence suggesting that sNfL could serve as a marker indicating the damaging effects of smoking on the brain and cognition.

sNfL is recommended as a serum biomarker for evaluating neuronal damage in the context of various diseases and assessing the effectiveness of drugs [37]. When neuronal cells are damaged due to various risk factors, NfL is released into the cerebrospinal fluid and eventually into the bloodstream [17]. sNfL levels are often regarded as “the neurologist’s C-reactive protein,” reflecting activity in certain nervous system diseases [38]. These diseases include Alzheimer’s disease, stroke, acute spinal cord injury, small vessel disease, neuromyelitis optica, head injury, human immunodeficiency virus (HIV) infection, multiple sclerosis, diabetic sensorimotor polyneuropathy, and Parkinson’s disease [17, 37, 39]. Some evidence suggests that serum NfL levels may be influenced by age, renal function, blood volume, and BMI [22, 23, 40]. Concerning lifestyle factors, previous studies have indicated an association between alcohol consumption and sNfL levels [41]. However, the existing literature on the relationship between tobacco smoking or cotinine and sNfL is limited. In a study that assessed the relationship between NfL levels and optical coherence tomography, active smokers exhibited higher NfL levels in participants with thin ganglion cell layer volume [42]. It is conceivable that inflammation may serve as a link between tobacco smoking and sNfL levels, with increased levels of circulating pro-inflammatory factors associated with smoking being correlated with sNfL levels.

Tobacco smoking has been linked to neural damage in the general population and has been shown to induce neurotoxic substances in the cerebrospinal fluid, contributing to neurogenerative injury processes [43]. Cotinine, a major biomarker of cigarette smoke exposure, is a metabolite of nicotine and a strong indicator of nicotine intake [44]. It is well-established that cotinine can affect the function of various cells and tissues, including those in the nervous system, by participating in oxidation-reduction processes. The brain, along with other organ systems, is highly susceptible to oxidative stress induced by cotinine and other tobacco metabolites, given its high energy and oxygen demands [45]. Additionally, cigarette smoking is associated with a depletion of antioxidants. In human studies, tobacco exposure has been linked to decreased levels of glutathione, serum superoxide dismutase, and vitamin C [46, 47]. Various biomarkers of brain burden and oxidative stress have shown different concentrations in smokers compared to non-smokers [48]. The interplay between cotinine and these nervous system biomarkers is undoubtedly multifaceted, and further research is warranted to explore these associations and elucidate the underlying pathological mechanisms.

In our investigation, we explored the relationship between smoke exposure and neurological function impairment in adults. Increased exposure to cigarettes may serve as an indicator of neurological function injury. Our findings demonstrated a positive correlation between the concentrations of serum cotinine and sNfL levels. This provides compelling evidence of the detrimental impact of cigarette smoking on neuronal integrity, degeneration, and neurocognitive function. Moreover, our subgroup analyses revealed that age and sex may be influential factors affecting the relationship between serum cotinine and sNfL levels. Notably, we observed a linear increase in sNfL levels with higher serum cotinine levels among individuals aged 60 years or older. Previous research has also highlighted the importance of age as a determinant of sNfL, with a positive correlation between age and sNfL, particularly in individuals over 60 years of age [23, 49]. Despite this, the existing literature on sex-related differences in sNfL remains limited. Our results suggest that sex may influence the clearance of sNfL, underscoring the need to consider sex as a relevant factor when interpreting this association.

Our study possesses several notable strengths that contribute to its validity and significance. The primary strength lies in the use of data from the NHANES, a comprehensive and nationally representative cross-sectional survey. This relatively large dataset enabled us to meticulously explore the intricate associations between tobacco smoke exposure and ln-transformed sNfL levels. Additionally, our study pioneers the investigation of cigarette smoking’s impact on biomarkers associated with the nervous system. This pioneering aspect of our research provides valuable insights into the deleterious effects of cigarette smoke and underscores the importance of smoking cessation.

However, as with any scientific study, our research is not without limitations, and these limitations should be acknowledged. Firstly, the cross-sectional design employed in our research inherently limits our ability to establish causal relationships. Although we have identified robust associations between serum cotinine levels and sNfL levels, it is crucial to emphasize that causation cannot be inferred from our findings. In acknowledging the constraints imposed by our study design, we recognize the importance of future research endeavors specifically aimed at exploring causal relationships in the context of smoke exposure and its impact on sNfL levels. Secondly, we must acknowledge the constraint imposed by the short half-life of cotinine. The observed results are reflective of short-term exposure levels, and they may not encapsulate the fluctuations in biomarker levels over a more extended period. This temporal limitation should be considered when interpreting our findings and highlights the need for future longitudinal studies that can capture changes in exposure and biomarker levels over time. Thirdly, we acknowledge the limitations associated with the assessment of smoking exposure through serum cotinine levels and self-reported questionnaires. These methods may introduce potential underestimation due to various confounding factors. Despite these constraints, our study strives to contribute valuable insights within the context of these acknowledged limitations. Lastly, our research, despite its methodological rigor, is constrained by the relatively limited sample size available for subgroup analyses. While our current analyses provide valuable insights, particularly in the context of subgroups, we recognize the need for caution when interpreting these specific results. Larger sample sizes in future studies could offer more robust and conclusive findings in these subgroups. Additionally, the utilization of research methods that enable the exploration of causality, such as prospective cohort studies or randomized controlled trials, is recommended.

## Conclusion

Our study has shown a significant and positive association between serum cotinine levels and sNfL levels in adult participants. Cigarette smoke exposure was found to be related to increased sNfL levels. This association underscores the potential neurotoxic effects of cigarette smoke, highlighting the importance of addressing and mitigating the impact of tobacco use on neurological health. Further research is warranted to provide additional evidence and expand our understanding of this relationship.

## Data Availability

Those datasets generated and analyzed in the current study can be found at NHANES website (https://www.cdc.gov/nchs/nhanes/index.htm). Accessed on 10 March 2023.
